# Sensor wide association studies in digital medicine

**DOI:** 10.1038/s41746-026-02821-0

**Published:** 2026-05-30

**Authors:** Nico Steckhan, Felix Broghammer, Dylan Powell

**Affiliations:** 1 Digital Health - Connected Healthcare, Hasso Plattner Institute, University of Potsdam, Potsdam, Germany; 2 Department for Prevention and Care of Diabetes, Department of Medicine III, Faculty of Medicine Carl Gustav Carus, Technische Universität Dresden, Dresden, Germany; 3 Federal Institute for Occupational Safety and Health, Dresden, Germany; 4 Faculty of Health Sciences & Sport, University of Stirling, Stirling, UK

**Keywords:** Computational biology and bioinformatics, Diseases, Health care, Medical research

## Abstract

Assoziation studies revolutionized genomics by rigorously screening large feature sets against health outcomes. Digital medicine now produces similarly high-dimensional, longitudinal sensor data from wearables, smartphones, and connected environments. We propose Sensor-Wide Association Studies (SWAS): structured, feature-wide, hypothesis-generating scans of a pre-specified library of sensor-derived features against one or more pre-defined clinical phenotypes, with transparent feature documentation, appropriate longitudinal modeling, and principled control of multiplicity. This perspective outlines minimal standards, common failure modes, and ethical considerations to help SWAS become a reproducible foundation for digital epidemiology and personalized medicine.

## From GWAS to SWAS: a natural progression

Genetic association studies introduced a data-driven method for uncovering links between the genome and various diseases or traits. GWAS, for example, scan millions of single nucleotide polymorphisms (SNPs) across large cohorts, identifying associations with conditions such as type 2 diabetes or cardiovascular disease. PheWAS takes a reverse approach: it begins with a single genetic variant and then looks across a wide array of phenotypes—encompassing anything from metabolic syndromes to mental health disorders—to see where the variant exerts measurable influence. Translating association signals into mechanism typically requires substantial downstream functional follow-up^1^.

Sensor-Wide Association Studies (SWAS) adapt and extend existing frameworks such as digital phenotyping^2^ and environment- or exposome-wide association studies (EWAS). Like EWAS, SWAS takes a systematic, agnostic approach, but the “exposures” here are continuous multimodal signals from wearable and IoT devices. Building on digital phenotyping, SWAS formalizes this data-driven paradigm into a structured analytic framework^3^, treating sensor-derived variables as candidates in large-scale association analyses with health outcomes. These streams, including heart rate, movement, sleep, respiratory rate, and beyond, yield digital biomarkers, defined as characteristics collected from digital health technologies that indicate biological processes (normal or pathogenic) or responses to interventions^4^. Unlike sporadic medical visits or self-report questionnaires, SWAS enables integration of granular, real-life sensor data with clinical endpoints. This structured approach offers the potential to reveal previously invisible patterns in human physiology and disease trajectories. By systematically assessing the dynamic behavior of multimodal data streams, SWAS can capture time-dependent features that more faithfully represent the “physiome” ^5^.

## The rise of sensor data

In the last decade, wearable devices such as smartwatches, fitness trackers, and even smart clothing have proliferated dramatically. One reason for their popularity is that they offer actionable insights into daily activities, like step counts, calorie estimates, heart rate trends, and sleep duration. Beyond mainstream consumer wearables, specialized medical-grade devices are also gaining traction, measuring parameters like glucose levels, electrocardiography (ECG), blood pressure, or continuous oxygen saturation. Wearable non-invasive sensors, such as smartwatches, smartphones, or rings, can externally record parameters like step count and heart rate; the second generation includes biochemically based sensors, while the third generation comprises wearables that actively intervene in the system, such as the artificial pancreas^6^. Often such devices are recording noninvasively and without users’ interaction. Simultaneously, the Internet of Things (IoT) has expanded data-collection possibilities beyond the body itself: smart thermostats, air-quality monitors, and location-tracking devices can supply context on an individual’s living environment.

This surge in sensor data parallels the emergence of large-scale biobanks and digital epidemiology initiatives. Studies such as the UK Biobank have begun incorporating continuous accelerometry, and projects like the U.S. “All of Us” Research Program plan to integrate wearable data for over a million participants. As these rich data sets grow in size, the natural next step is to develop systematic methods, reminiscent of GWAS or PheWAS, but geared toward continuous and frequently high-dimensional sensor data. Large scale wearable studies have shown associations of physical activity and diabetes mortality, and a spectrum of various diseases categories^7–9^. Further multimodal sensors might enable a wider application in a variety of contexts, such as the development of neuro, immune or endocrine digital biomarkers. Recently, a protocol for the modeling of a biomarker for inflammation has been proposed ^10^.

A couple of digital epidemiology studies have been developed during the COVID-19 pandemic^11–14^. The German Corona Data Donation has been a milestone to capture wearable data on a public health level in the European Union^14^. Over nearly three years, the Corona Data Donation project gathered wearable data from more than 190,000 monthly active resident participants to support the detection of COVID-19 and investigate the long-term effects of SARS-CoV-2 infection. Providing further insights into fever curves, activity and sleep patterns and circadian rhythms of thousands of people stratified by their location. Combining environmental sensors (e.g., meteorological data) with health sensors represents even more potential analyses as shown in such cohorts.

## Defining SWAS

**Fig. 1:**
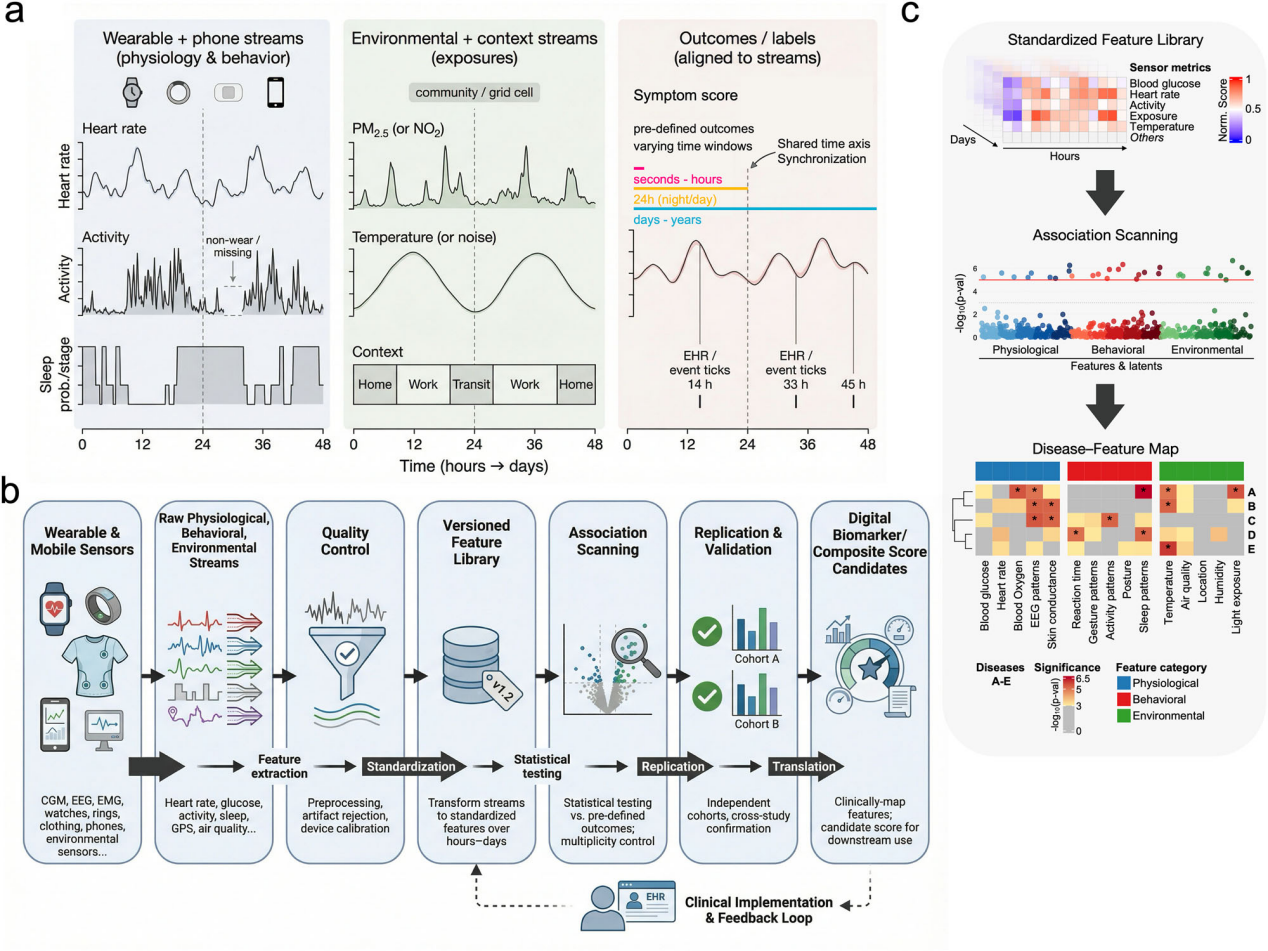
End-to-end concept for Sensor-Wide Association Studies (SWAS). **a** Multi-modal data streams from wearable and mobile sensors. Physiological and behavioral streams (left) — including heart rate, physical activity, and sleep staging — are recorded alongside environmental and contextual exposures (center), such as particulate matter (PM₂.₅), temperature, noise, and location context. All streams are aligned to a shared time axis and synchronized with clinical outcomes and event labels (right), which span time windows from seconds–hours to days–years, including symptom scores, pre-defined endpoints, and electronic health record (EHR) event ticks. **b** SWAS analysis pipeline. Raw signals from diverse sensor modalities (CGM, EEG, EMG, watches, rings, smart clothing, phones, environmental sensors) undergo feature extraction, preprocessing, artifact rejection, and device calibration (Quality Control), then are transformed into a versioned, standardized feature library across hours-to-days time scales. Features are systematically scanned against pre-defined outcomes with multiplicity control (Association Scanning), replicated in independent cohorts with cross-study confirmation (Replication & Validation), and translated into candidate digital biomarkers or composite scores for downstream clinical use. A clinical implementation and feedback loop connects validated outputs back to EHR systems. **c** Illustrative discovery output. A normalized sensor-metric heatmap (top, “Versioned Feature Library”) summarizes multi-modal feature behavior over hours and days. Association scanning (middle, “Association Scanning”) yields feature-level significance profiles (−log₁₀ p-value) across physiological, behavioral, and environmental categories. Validated associations (bottom, “Biomarker Map”) are mapped across diseases (A–E), highlighting disease-specific digital biomarker signatures with significance encoded by color intensity. (CGM continuous glucose monitor, EEG electroencephalography, EMG electromyography, GPS global positioning system, EHR electronic health record).

A SWAS starts by collecting (or accessing) a broad palette of sensor streams from a large cohort of participants over a defined period—potentially weeks, months, or even years. Each sensor modality is processed in order to engineer features, often creating a massive matrix of variables. For instance, a single wearable might log heart rate every minute, step counts per hour, sleep stages overnight, and more. Combined with other contextual data (e.g., ambient temperature, local air pollution indices, or even phone usage patterns), the resulting feature set can be extremely high-dimensional (Fig. 1a).

Next, investigators pre-specify the outcome(s), key covariates, and the unit of analysis (e.g., person-level summaries over fixed time windows, event-centered segments, or repeated daily windows). A SWAS then systematically tests associations between a large, pre-defined library of sensor-derived features and one or more pre-defined outcomes, using models that respect within-person correlation and longitudinal structure. Because feature libraries are ad hoc and strongly correlated, SWAS should explicitly justify its error-control strategy (e.g., false discovery rate control via Benjamini-Hochberg, q-value estimation, permutation-based thresholds, or knockoff-style procedures). Putative associations should be interpreted as hypothesis-generating and treated as candidate digital biomarkers until they replicate in independent data sets and, ideally, across device ecosystems (Fig. 1b). Downstream, replicated features can be summarized into composite profiles (e.g., a digital biomarker fingerprint) or combined into Poly-Sensor Risk Scores, which must be evaluated for calibration, net benefit, and transportability before clinical use ^15^.

## Operational definition and minimal standards

### Operational definition

SWAS is a feature-wide association scan in which a pre-specified library of sensor-derived features is systematically tested against one or more pre-defined clinical phenotypes in a cohort (or discovery cohort), with transparent feature documentation, appropriate longitudinal modeling, and explicit error control.

A study should only claim the SWAS label if it includes, at minimum:

- Pre-specified outcomes, key covariates, and a stated unit of analysis before large-scale scanning.
- Transparent feature library documentation (definition, time window, preprocessing, units, missingness handling) to enable reuse and auditability.
- Statistical models that respect person-level clustering and repeated measures and report effect sizes with uncertainty, not only p-values.
- Reported and justified multiplicity control for the chosen feature space (e.g., FDR, permutation, knockoffs).
- Validation or replication where feasible (e.g., split-sample, external cohort, or cross-device replication).

## Where the GWAS analogy breaks down

Unlike genotypes, sensor-derived features are mutable, context- and device-dependent, and often downstream of health status, behavior, or interventions. These asymmetries make SWAS especially vulnerable to reverse causation and dense confounding (e.g., socioeconomic status, baseline health, seasonality, and engagement with technology). Consequently, SWAS findings should not be interpreted as GWAS-like causal effects; they are primarily associative and hypothesis-generating signals that require triangulation.

Rigor in SWAS therefore, depends on temporal design (e.g., lagged features, event-centered windows), rich contextual covariates, sensitivity analyses (negative controls, stratification by device and adherence), and replication across cohorts and device ecosystems. When causal claims are desired, SWAS should be paired with explicit causal-inference designs rather than relying on the breadth of scanning alone.

## Proposed SWAS workflow

We propose a minimal, reproducible SWAS pipeline (Fig. 1b) that mirrors the discipline of large-scale association studies while accounting for the realities of longitudinal sensor data:

1. **Cohort and phenotype specification:** Define eligibility, follow-up, pre-specified outcome(s), key covariates, and the unit of analysis (windows/segments).
2. **Data ingestion and synchronization:** Harmonize multimodal streams from wearable and mobile sensors—including CGM, EEG, EMG, watches, rings, smart clothing, phones, and environmental sensors—and align raw physiological, behavioral, and environmental streams in time with contextual metadata (Fig. 1b, *Feature Extraction*).
3. **Preprocessing and quality control:** Handle artifacts, non-wear, missingness (often informative), device heterogeneity, and long-term drift or firmware changes through systematic preprocessing, artifact rejection, and device calibration (Fig. 1b, *Quality Control*).
4. **Feature engineering:** Compute a pre-defined feature library across multiple temporal windows (hours to days); document algorithms, units, and versioning to ensure reproducibility (Fig. 1b, *Versioned Feature Library / Standardization*).
5. **Association scanning:** Systematically test standardized features against pre-defined outcomes with multiplicity control, including FDR, permutation, or knockoff-based corrections, and run sensitivity checks such as negative controls, lagged analyses, and stratification (Fig. 1b, *Association Scanning / Statistical Testing*).
6. **Replication and validation:** Replicate significant associations in independent cohorts with cross-study confirmation, and assess transportability across devices and populations (Fig. 1b, *Replication & Validation*).
7. **Translation:** Clinically map validated features and derive candidate digital biomarkers, composite scores, or Poly-Sensor Risk Scores for downstream use, supported by a clinical implementation and feedback loop connecting back to EHR systems (Fig. 1b, *Translation / Digital Biomarker Candidates*).

## Potential applications

SWAS is primarily a discovery and hypothesis-generation framework. The applications below should be interpreted as downstream uses of rigorously conducted SWAS, in which individual features (or derived composites) are treated as candidate digital biomarkers until replicated across cohorts, device ecosystems, and populations and evaluated for calibration, clinical utility, and transportability.

### Early disease detection

SWAS can surface candidate sensor features or feature clusters that consistently change before clinically recognized events (e.g., infection onset or metabolic decompensation). Infections, metabolic dysregulation, or inflammatory processes often leave subtle physiological signatures before symptoms appear, and prior wearable studies suggest that shifts in resting heart rate, skin temperature, and sleep efficiency can be informative^2^. In a SWAS framework, such signals are identified systematically across a broad feature library and then prioritized for replication and prospective evaluation before being proposed as digital biomarkers or composite “fingerprints.”

### Chronic disease management

For individuals living with chronic conditions (e.g., heart failure, diabetes, COPD), continuous monitoring can enable earlier detection of exacerbations and assessment of treatment response. SWAS can help identify multi-signal composites (for example, heart-rate variability, activity variability, and environmental context) that predict decompensation. However, such composites should be treated as candidate predictors until they have been replicated across cohorts and devices and evaluated with appropriate clinical-performance metrics (calibration, net benefit, and stability over time).

### Mental health monitoring

Mental health disorders are notoriously underdiagnosed and undertreated due to social stigma, limited access to specialized services, and a lack of objective biomarkers. SWAS could help identify reproducible associations between physiological or behavioral patterns (e.g., disrupted circadian rhythms, diminished activity variability, and abnormal sleep patterns) and validated clinical ratings for conditions such as major depression or anxiety disorders^14^. Because behavioral features are tightly coupled to context and self-management, results should be interpreted cautiously and validated across settings and populations before informing intervention thresholds.

### Lifestyle and population health

Large-scale SWAS efforts could clarify how daily behaviors (physical activity, sleep, social rhythms) and environmental exposures shape disease risk at population scale. Location- or community-level sensor data (e.g., local pollution, noise, temperature) can help identify modifiable drivers of exacerbations and inform public health interventions. Integrating real-time environmental data with exposome intelligence has been proposed as a route to more proactive risk assessment^16^, but SWAS results must still be checked for confounding by geography, seasonality, and socioeconomic factors.

### Additional application areas

In sports medicine, occupational health, and rehabilitation, SWAS can link diverse sensor signals (biomechanics, heart rate variability, workload, environment) to injury risk, recovery trajectories, and functional outcomes. These domains are well suited for SWAS because outcomes can often be defined prospectively, enabling clearer temporal ordering and stronger validation designs.

## Methodological challenges

### Data quality and standardization

Wearable data can be noisy, incomplete, and inconsistent across devices. Even within a single cohort, participants may use different brands or models, each with proprietary filtering and calibration. A SWAS should therefore report device types, firmware/algorithm versions when available, and implement quality control for artifacts, non-wear, and implausible values, alongside harmonization or stratification across devices. Practical lessons and reporting frameworks from digital biomarker development can be used to standardize these steps ^15,17^.

### Contextual dependency

Sensor features are highly context-dependent: a spike in heart rate may reflect exercise, stress, dehydration, medication effects, or measurement error. Disentangling contexts requires auxiliary metadata (activity labels, location, schedule/season, self-report, smartphone logs) and careful temporal alignment. Without context, SWAS can produce spurious associations or miss genuine ones; negative-control outcomes and stratified analyses can help detect residual confounding.

### Statistical and computational complexity

SWAS entails multiple testing on a vast scale, often with highly correlated features. Unlike GWAS, there is no meaningful universal significance threshold because SWAS feature libraries are cohort- and device-specific and can vary widely in size and dependence structure. Instead of relying on a GWAS-style threshold by analogy, SWAS should (i) define and version the feature space, and (ii) control and report false discoveries using strategies appropriate for dependence, such as false discovery rate control (Benjamini-Hochberg), q-values, permutation-based thresholds, or knockoff-style procedures. Importantly, broad feature engineering is still a form of hypothesis encoding; SWAS is best described as hypothesis-generating, not hypothesis-free.

SWAS analyses should explicitly address longitudinal structure and time-varying confounding. Within-person correlation can be handled using mixed models or generalized estimating equations^18,19^, and survival models with time-varying covariates^20^. Because non-wear and missingness can be informative, SWAS should avoid default listwise deletion and instead use principled approaches such as multiple imputation or inverse-probability weighting^21^. When causal interpretations are desired, SWAS findings should be paired with explicit causal-inference strategies for time-varying exposures, such as marginal structural models^22^. Temporal dependencies and drift can be modeled using state-space approaches or recurrent architectures ^23,24^.

### Privacy and ethical considerations

Continuous sensor monitoring captures intimate details about daily life and can reveal habits, locations, and social interactions. Research institutions must implement privacy-by-design (data minimization, de-identification, secure enclaves) and ensure that data use aligns with meaningful, revocable consent and governance models, including emerging approaches to citizen data sovereignty^25^. Beyond privacy, SWAS must address representativeness and equity: device ownership, digital literacy, and connectivity vary by age, socioeconomic status, and geography, creating biased sampling and unequal benefit distribution. Commercial platform dependence is another core risk: access to raw data may be restricted, and vendor algorithms or firmware can change without notice, altering feature meaning over time. SWAS reports should therefore document device and algorithm versions, quantify differential missingness, and include fairness-aware analyses and community-informed governance where possible.

## The road ahead

Sensor-Wide Association Studies provide a disciplined way to interrogate the rapidly expanding universe of longitudinal sensor features against clinically meaningful outcomes. By borrowing the transparency and scale of genetic association studies while adapting to sensor-specific pitfalls, SWAS can help prioritize candidate digital biomarkers for replication and prospective evaluation.

For SWAS to be credible, investigators should adhere to minimal standards: pre-specify outcomes and feature libraries, document preprocessing and device versions, use longitudinal models with appropriate multiplicity control, and treat discoveries as provisional until replicated and stress-tested for confounding, adherence effects, and transportability. Journals and reviewers can accelerate the field by expecting explicit reporting checklists and replication plans when the SWAS label is used.

Progress will also require shared, versioned sensor feature libraries, open-source tooling for quality control and harmonization, and governance models that protect privacy while enabling responsible reuse. With these ingredients, SWAS can bridge data abundance and clinical relevance - not by promising GWAS-like causal certainty, but by offering a reproducible pathway from broad association scans to validated, actionable digital biomarkers.
